# Pesticidal and pest repellency activities of a plant derived triterpenoid 2α,3β,21β,23,28-penta hydroxyl 12-oleanene against *Tribolium castaneum*

**DOI:** 10.1186/0717-6287-47-68

**Published:** 2014-12-15

**Authors:** Alam Khan, Md Shariful Islam, Moizur Rahman, Tanjeena Zaman, Md Ekramul Haque

**Affiliations:** Department of Pharmacy, University of Rajshahi, Rajshahi, 6205 Bangladesh; Department of Animal Husbandry and Veterinary Science, University of Rajshahi, Rajshahi, 6205 Bangladesh; Department of Fisheries, University of Rajshahi, Rajshahi, 6205 Bangladesh

**Keywords:** *Laportea crenulata*, 2α,3β,21β,23,28-penta hydroxyl 12-oleanene, Botanical pesticide, Botanical pest repellent, *Tribolium castaneum*

## Abstract

**Background:**

*Tribolium castaneum* (Herbst) is a major pest of stored grain-based products, and cause severe damage to cereal grains throughout the world. The present investigation was aimed to determine the pesticidal and pest repellent activities of 2α,3β,21β,23,28-penta hydroxyl 12-oleanene against *T. castaneum*. The compound 2α,3β,21β,23,28-penta hydroxyl 12-oleanene is a triterpenoid which was isolated from the roots of *Laportea crenulata* Gaud. Surface film technique was used for pesticidal screening, whereas, pest repellency property of the triterpenoid was determined by filter paper disc method.

**Results:**

At 24 hours of exposure duration, significant mortality records (80% and 86%) were observed at doses 0.88 and 1.77 mg/cm^2^. No significant change in mortality records was observed when duration of exposure was increased up to 48 hours. The triterpenoid showed significant repellency activity at doses 0.47 and 0.94 mg/cm^2^.

**Conclusion:**

These data suggest that the triterpenoid 2α,3β,21β,23,28-penta hydroxyl 12-oleanene possess both pesticidal and pest repellency activities against *T. castaneum* and can be used in controlling the pest of grain-based products.

**Electronic supplementary material:**

The online version of this article (doi:10.1186/0717-6287-47-68) contains supplementary material, which is available to authorized users.

## Background

The red flour beetle, *Tribolium castaneum* (Herbst) is a primary pest of flour and other milled products of cereals, and a secondary pest of stored wheat, causing severe damages to these food grains by both quantity and quality [1–4]. A number of synthetic agents (e.g. methoprene, permethrin, cypermethrin, deltamethrin and fenvalerate etc.) were identified for good activity against *T. castaneum* and have been used to control the pest [4, 5]. However, use of synthetic agents has led to a number of problems such as environmental disturbances, increasing costs of application, pest resurgence, pest resistance to pesticides, lethal effects on non-target organisms and direct toxicity to users [6]. Dyte and Blackman ([7]) reported that almost all of the strains of *T. castaneum* have become resistant to malathion. In recent years, there has been increasing information on the use of alternative methods [8], and plant extracts are the most commonly tested alternative products [9–14]. Different biological activities of plant derivatives were demonstrated for the control of stored-grain pests [11, 15]. Plant products having considerable insecticidal potential are gaining remarkable importance in recent years because of minimizing the disadvantages associated with synthetic agents [16, 17]. Certain plant families, particularly plant products of Rutaceae and Myrtaceae had shown, in previous observations, repellent, insecticidal, anti-feedant, and growth regulatory properties against insect pests of stored commodities [18–24]. Some of the Citrus plant species (*C. sinensis, C. aurantifolia, C. reticulata, C. limon*) (Rutaceae) have been also reported as a source of botanical insecticides [24–26]. A variety of these plants contain secondary metabolites that show insecticidal activity against several coleopteran and dipterans species [27, 28].

*Laportea crenulata* Gaud. (Synonym *Urtica crenulata* Roxb.) is an evergreen shrub of Urticaceae family [29]. The *Urticaceae* are monoecious or dioecious herbs or infrequently shrubs or small trees comprising 45 genera and 700 species, often with specialized stinging hairs [30]. About 9 genera and 60 species are available in Bangladesh [31]. In Bangladesh, *L. crenulata* locally known as Agnichutra, also distributed in India and Malay island [29]. The roots are used traditionally for the treatment of bleeding from nose and/or mouth, excessive gas in the stomach, constipation, weakness, asthma, gout, mumps, whooping cough, and chronic fever [32]. The roots of the plant have stimulant, stomachic and diuretic properties [32]. Such traditional uses suggesting the presence of biologically active substances in roots of the plant. A triterpenoid 2α,3β,21β,23,28 − penta hydroxyl 12-oleanene was isolated from the roots of the plant [33]. In searching botanical agent(s) to control *T. castaneum*, the present study was aimed to determine the insecticidal and insect repellency activities of 2α,3β,21β,23,28-penta hydroxyl 12-oleanene against *T. castaneum*.

## Results

**Figure 1:**
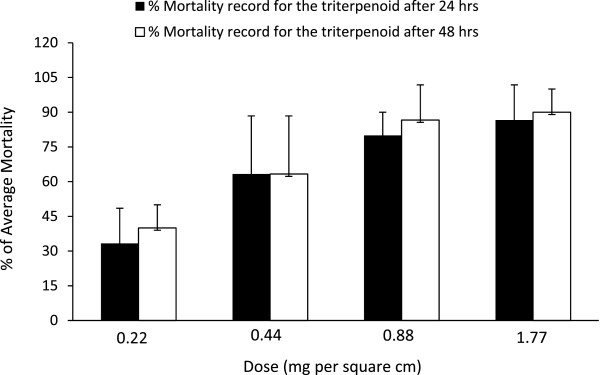
**Mortality records of 2α,3β,21β,23,28-penta hydroxyl 12-oleanene (triterpenoid) after 24 and 48 hours.** Average mortality records were increased with dose of the triterpenoid. On increasing the duration of the triterpenoid exposure to the pest, the mortality records were not significantly varied. Standard deviations are shown as error bar at the top of each column.

The compound 2α,3β,21β,23,28-penta hydroxyl 12-oleanene at 24 hours duration of exposure, has exerted significantly high mortality records 80.00% and 86.60% at doses 0.88 and 1.77 mg/cm^2^, respectively (Table 1; Figure 1). The compound showed moderate mortality (63.30%) at dose 0.44 mg/cm^2^ and weak mortality (33.30%) at dose 0.22 mg/cm^2^; both were not significant for pesticidal uses. The higher mortality records with higher doses of the compound suggesting its dose dependent pesticidal activity against *T. castaneum*. When exposure duration was increased 24 to 48 hours, the mortality records were minutely increased for doses 0.22, 0.88 and 1.77 mg/cm^2^, however, for 0.44 mg/cm^2^ dose no change in mortality was observed (Table 2; Figure 1). Median lethal doses (LD_50_) of the compound for 24 and 48 hours exposure duration were 0.38 and 0.34 mg/cm^2^, respectively (Table 1).

**Table 1:** **LD**_**50**_ **calculation for pesticidal activity using probit analysis**

Recording time	Dose (mg/cm ^2^)	% of Average mortality	% Crrected mortality	Regression equation	LD _50_ (mg/cm ^2^)	95% Confidence limits	
						Lower	Upper
Record after 24 hours	1.77	86.60	87	Esimate 1	0.38	6.58	16.32
				Y = 2.15 + 3.42X			
	0.88	80.00	80	Esimate 2			
	0.44	63.30	63	Y = 1.92 + 1.58X			
	0.22	33.30	33				
Record after 48 hours	1.77	90.00	90	Esimate 1	0.34	9.54	22.55
	0.88	86.60	87	Y = 1.62 + 1.64X			
	0.44	63.30	63	Esimate 2			
	0.22	40.00	40	Y = 3.23 + 1.31X

**Table 2:** **Screening of pesticidal activity for 2α,3β,21β,23,28-penta hydroxyl 12-oleanene**

Dose (mg/cm ^2^)	#	Mortality record for applied pests			
		Record after 24 hours	Average ± SD record after 24 hours	Record after 48 hours	Average ± SD record after 48 hours
1.77	10	7	8.66 ± 1.52	8	9.00 ± 1.00
1.77	10	9		9	
1.77	10	10		10	
0.88	10	8	8.00 ± 1.00	10	8.66 ± 1.52
0.88	10	9		9	
0.88	10	7		7	
0.44	10	9	6.33 ± 2.51	9	6.33 ± 2.51
0.44	10	6		6	
0.44	10	4		4	
0.22	10	2	3.33 ± 1.52	4	4.00 ± 1.00
0.22	10	5		5	
0.22	10	3		3	
Control	10	0	0	0	0

# = Number of pests applied per petridish.

At the beginning of exposure (1 h), moderate pest repellency activity (53.2 and 66.6% repulsion) was observed for doses 0.47 and 0.94 mg/cm^2^, respectively (Table 3; Figure 2). Within the second hour, repellency records were sufficiently increased for all doses. At subsequent 3rd and 4th hours observation repellency records were little increased, but no increment was observed after 4th hour (Table 3). However, repellency activity at the lowest dose 0.23 mg/cm^2^ always found insufficient. Overall, significant pest repellency activity for the triterpenoid was observed at 0.47 and 0.94 mg/cm^2^.

**Table 3:** **Pest repellency records and percent repulsions (PR) of 2α,3β,21β,23,28-penta hydroxyl 12-oleanene**

Dose (mg/cm ^2^)	#	Repellency record														
		Hourly observations					Average of hourly observations (Nc)					Percent repulsion (PR) PR = (Nc-5)×20%				
		1 h	2 h	3 h	4 h	5 h	1 h	2 h	3 h	4 h	5 h	1 h	2 h	3 h	4 h	5 h
0.94	10	8	8	8	9	9	8.33	9.00	9.00	9.66	9.66	66.6%	80.0%	80.0%	93.2%	93.2%
0.94	10	7	9	9	10	10										
0.94	10	10	10	10	10	10										
0.47	10	8	9	9	9	9	7.66	8.00	8.33	8.66	8.66	53.2%	60.0%	66.6%	73.2%	73.2%
0.47	10	7	7	8	8	8										
0.47	10	8	8	8	9	9										
0.23	10	6	6	6	6	6	6.33	7.00	7.33	7.33	7.33	26.6%	40.0%	46.6%	46.6%	46.6%
0.23	10	5	7	8	8	8										
0.23	10	8	8	8	8	8										

# = Number of pests applied per petridish.

**Figure 2:**
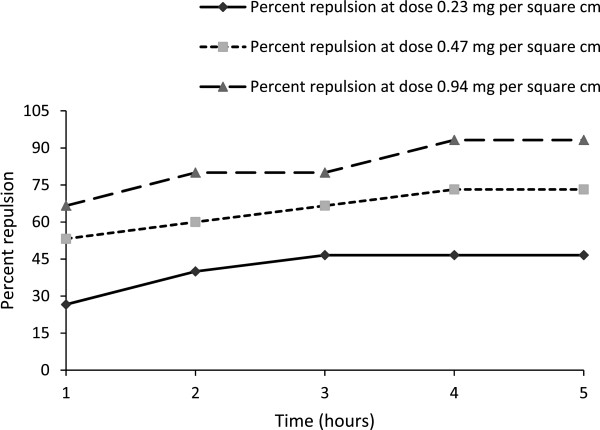
**Repellency records of 2α,3β,21β,23,28-penta hydroxyl 12-oleanene per hour interval up to 5 hours.** With doses of 2α,3β,21β,23,28-penta hydroxyl 12-oleanene, the repellency property (percent repulsion) against the pest was increased. As duration of exposure of the compound toward the pest was increased, the repellency property was increased up to 4 h of exposure, and after that it became constant.

## Discussion

Insects/Pests are a problem in stored grain throughout the world, and cause serious losses in weight and quality of the stored products [17, 34]. *T. castaneum* is a major paste of most grain, widely spread worldwide and very destructive [5, 16]. As widely used synthetic insecticides/pesticides causing concern because of environmental, economy, resistance and toxicity issues, scientists are searching for alternative methods [6, 8]. In many regions of the world, locally available materials are used to protect stored products against damage by pest infestation. Insecticides of plant origin, because of their high degree of tolerance by the mammals, are particularly desired for application against pests of fodders, fruits, vegetables and stored grains [35]. Moreover, interest in botanical insecticides has increased as a result of environmental concerns, and insect populations becoming resistant to conventional chemicals. The using of plant extracts in insect control has been practiced for at least two millennia, when botanical insecticides were considered important products for insect management in Ancient China, Egypt, India, Greece [5, 6, 36] *.* Moharrammipour et al. [37] and Shahkarami et al. [38] demonstrated that *Ferula asafetida* L. extract and the essential oil of *Artemisia aucheri* Boiss. had antifeedant property on *T. castaneum* adults.

Triterpenoid 2α,3β,21β,23,28-penta hydroxyl 12-oleanene was isolated from roots of *L. crenulata*, has been shown both insecticidal and insect repellency properties against *T. castaneum*. The control of pest in stored food products can be achieved by insecticidal and insect repellency activities [4, 12, 16, 39]. Excellent pesticidal activity for the triterpenoid has been found at doses 0.88 and 1.77 mg/cm^2^, whereas, sufficient pest repellency activity was observed at doses 0.47 and 0.94 mg/cm^2^. Identification of pesticidal compounds in plant is not new; Luo et al. [40] isolated triptolide, triptonide and euonine from root barks of *Tripterygium wilfordii* Hook and confirmed activity against *Mythimna separate*. Pungitore et al. [41] identified oleanolic acid, maslinic acid, and daucosterol from *Junellia aspera* (Verbenaceae) and confirmed their toxic effect against *T. castaneum*. However, only a small number of pest control products directly obtained from plants [19, 42]. Botanicals used as insecticides presently constitute only 1% of the world insecticide market [43]. In the present study, we confirmed toxic effect of the triterpenoid against *T. castaneum*. However, further investigations are necessary to know toxicity of the compound to mammals as well as to know the effect on nutritional status of grain.

## Conclusions

The compound 2α,3β,21β,23,28-penta hydroxyl 12-oleanene has induced significant pesticidal activity against *T. castaneum* at doses 0.88 and 1.77 mg/cm^2^. In contrast, significant pest repellency against the pest was observed at doses 0.47 and 0.94 mg/cm^2^. In the light of the findings of present study, it can be stated that good pesticidal and pest repellency activities of 2α,3β,21β,23,28-penta hydroxyl 12-oleanene against *T. castaneum* suggesting its suitability as botanical pesticide in controlling the pest of stored grains and grain products.

## Methods

### Plant materials

The roots of *L. crenulata* was collected from various part of Rangpur district of Bangladesh in the month of October to November. Taxonomically the plant was identified by Professor A. T. M. Naderuzzaman, Department of Botany, University of Rajshahi, Rajshahi, Bangladesh and a voucher specimen (No. 1239) had been deposited in the Department.

The fresh roots was first washed with water to remove adhering dirt, cut into small pieces, sun dried for three days and finally dried at 45°C for 36 h in an electrical oven [33]. The dried roots were pulverized into a coarse powder [44] with the help of a grinding machine (FFc-15, China) and were stored in an air tight container for further use.

### Isolation of 2α,3β,21β,23,28-penta hydroxyl 12-oleanene

Dried powdered roots (900 g) of the plant were extracted (cold) with ethanol (5 L) in flat bottom glass containers, through occasional shaking and stirring for 10 days. The whole extract was filtered and the solvent was evaporated to dryness *in vacuo* with an rotary evaporator at 40–50°C to afford a blackish green mass (45 g), which was further extracted with petroleum ether (3×50 mL), chloroform (3×50 mL), and methanol (3×50 mL) to afford petroleum ether (3 g), chloroform (7 g), and methanol (9 g) fractions, respectively [45, 46].

**Figure 3:**
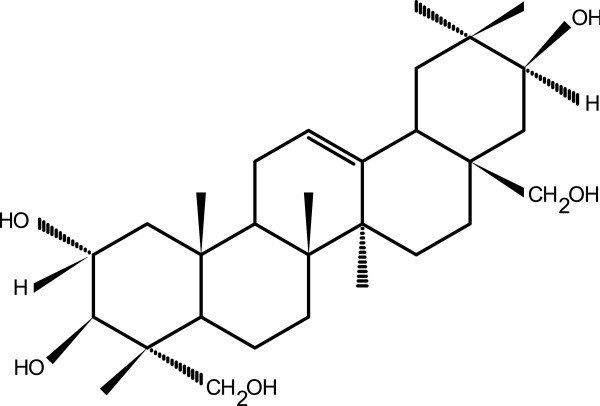
**Structure of compound 1 (2α,3β,21β,23,28-penta hydroxyl 12-oleanene).** The compound has five functional (hydroxyl) groups at 2, 3, 21, 23 and 28 positions, and one unsaturation (double bond) at 12 position.

The petroleum ether soluble fraction (5 g) was subjected to column chromatography using n-hexane, chloroform and methanol of increasing polarity. Column chromatography fractions eluting with 100% chloroform to 40% methanol in chloroform was subjected to preparative TLC (Silica gel PF254) with solvent system ethyl acetate: cyclohexane (2 : 1) to afford compound 1. Using mass spectroscopy (HR/ES-MS), IR, ^1^H-NMR, ^13^C-NMR, COSY, HSQC, HMBC, JMOD and NOESY the compound 1 was identified as a new triterpenoid 2α,3β,21β,23,28-penta hydroxyl 12-oleanene (Figure 3) [29, 33].

### Collection and maintenance of pest

The pest *T. castaneum* (Herbst) was originally collected from the Crop Protection Department of the University of Newcastle, U.K., and were maintained in the Crop Protection and Toxicology Laboratory of Department of Zoology, University of Rajshahi, Bangladesh. *T. castaneum* was reared in 1 L glass jar containing food medium, and a filter paper was placed inside each jar for easy movement of the pest. The jar was covered with a filter paper at the top, and kept in an incubator at 30 ± 0.5°C.

As food medium wheat flour and powdered brewers yeast in the ratio of 19 : 1 was used to culture the pest. Both flour and yeast were previously passed through a 250 micrometer aperture sieve and mixed thoroughly using an electric blender. The food was sterilized in an oven at 120°C for 6 h. Food was not used until at least 15 d after sterilization to allow its moisture content to equilibrate with that of environment.

### Screening for insecticidal activity

Screening of insecticidal activity was carried out by surface film method [4, 39, 47]. The working solution was prepared by dissolving 100 mg experimental sample in 2 ml mixed solvent (50% chloroform + 50% methanol) in a vial. For each sample similar three vials were prepared.

Thirteen clean and dried petridishes (size of each is 60 mm, area of each is 28.26 cm^2^) were taken for each sample. Four petridishes were marked by 50 mg, 25 mg, 12.5 mg and 6.25 mg. One ml working solution was poured into the 50 mg petridish and agitated clockwise, anticlockwise, left to right and right to left to further confirm the uniform dispersion, then 1 ml solvent (50% chloroform + 50% methanol) was added to that vial from which 1 ml had been used and mixed uniformly. From this vial, 1 ml solution was poured into the 25 mg petridish and agitated similarly for uniform dispersion. Using this serial dilution technique, likewise sample was poured into 12.5 mg and 6.25 mg petridishes and agitated similarly for uniform dispersion. The above processes were continued two times further using two remaining vials of working solution and eight remaining petridishes. Then the layers of dispersed sample into the petridishes were air dried. Further, 1 ml solvent (50%chloroform + 50%methanol) was poured and dispersed into control petridish and air dried.

The insects were collected by sieving and ten insects were applied on each layer of dispersed sample into the petridish. This process is continued for each petridish. Then the numbers of dead insects were recorded after passing 24 and 48 h.

### Insect repellency test

Filter paper disc method [4, 16, 17, 39, 48] was used to determine repellency property of the triterpenoid. The working solution was prepared by dissolving 60 mg experimental sample in 2 ml mixed solvent (50% chloroform + 50% methanol) in a vial. For each sample similar three vials were prepared.

Nine clean and dried petridishes (size of each is 90 mm) and Nine filter papers (size-90 mm) were taken for each sample. Three petridishes were marked by 30 mg, 15 mg and 7.5 mg. Three filter papers were taken for these three petridishes and each filter paper was cut (by scissors) into equal two parts through centre, where one part can be used as control part and other part can be used as treated part. For 30 mg petridish with its filter paper, treated part of filter paper was taken at outer background of the petridish and 1 ml working solution (prepared previously) was dispersed uniformly thorough out this part of filter paper and air dried. Then this part of filter paper was joined with its control part using transparent adhesive tape and placed into the 30 mg petridish using forceps. For 15 mg petridish with its filter paper, treated part of filter paper was taken at outer background of 15 mg petridish. One ml solvent (50% chloroform + 50% methanol) was added to that vial from which 1 ml had been used and mixed uniformly. From this vial, 1 ml solution was dispersed uniformly throughout the treated part of filter paper and air dried. Then this part of filter paper was joined with its control part using transparent adhesive tape and placed into the 15 mg petridish using forcep. Similar works was done for 7.5 mg petridish with its filter paper. The above processes were continued two times further using two remaining vials of working solution and six remaining petridishes and filter papers.

Ten insects were applied at the center of each filter paper that present in the petridish. This process was continued for each petridish. Then the number of insects have repelled were counted per hour interval up to 5 h. The percentages of repellency were determined and results were analyzed through ANOVA after transforming them into arcsin percentage value.

### Statistical analysis

The mortality data were subjected to probit analyses using SPSS (2001) to estimate LD_50_ of the compound against the stored product insect *T. castaneum*. The repellency data were calculated for percent repellency, which was again transformed using arcsine transformation for the calculation of analysis of variances (ANOVA). Mean values were compared using one way ANOVA (two factors without replication) (Additional file 1: Table S1).

## Electronic supplementary material

Additional file 1: Table S1: Two factors ANOVA (without replication) for repellency records through Arcsin transformation data. (DOCX 16 KB)
