# Supplementary material for: Pesticidal and pest repellency activities of a plant derived triterpenoid 2α,3β,21β,23,28-penta hydroxyl 12-oleanene against Tribolium castaneum
Source: Biol Res. 2014 Dec 15;47(1):68. doi: 10.1186/0717-6287-47-68 (PMC4289592; doi:10.1186/0717-6287-47-68)
Supplement: Supplementary file 1 — Additional file 1: Table S1: Two factors ANOVA (without replication) for repellency records through Arcsin transformation data. (DOCX 16 KB) [file 40659_2014_56_MOESM1_ESM.docx]

**Additional file 1: Table S1 Two factors ANOVA (without replication) for repellency records through Arcsin transformation data.**

| Source of Variations | SS | df | MS | F-ratio | P-value |
| --- | --- | --- | --- | --- | --- |
| Between dose levels | 1262.48 | 2 | 662.42 | 31.66 | 0.00063 |
| Between time intervals | 1032.32 | 4 | 496.32 | 17.64 | 0.00344 |
| Error | 172.53 | 8 | 24.62 |  |  |
| Total | 2467.34 | 14 |  |  |  |
